# Understanding the neuroinflammatory response following concussion to develop treatment strategies

**DOI:** 10.3389/fncel.2012.00058

**Published:** 2012-12-12

**Authors:** Zachary R. Patterson, Matthew R. Holahan

**Affiliations:** Department of Neuroscience, Carleton UniversityOttawa, ON, Canada

**Keywords:** neuroinflammation, concussion, mTBI, IL-10, IL-1, TNF-alpha, TGF-beta, IL-6

## Abstract

Mild traumatic brain injuries (mTBI) have been associated with long-term cognitive deficits relating to trauma-induced neurodegeneration. These long-term deficits include impaired memory and attention, changes in executive function, emotional instability, and sensorimotor deficits. Furthermore, individuals with concussions show a high co-morbidity with a host of psychiatric illnesses (e.g., depression, anxiety, addiction) and dementia. The neurological damage seen in mTBI patients is the result of the impact forces and mechanical injury, followed by a delayed neuroimmune response that can last hours, days, and even months after the injury. As part of the neuroimmune response, a cascade of pro- and anti-inflammatory cytokines are released and can be detected at the site of injury as well as subcortical, and often contralateral, regions. It has been suggested that the delayed neuroinflammatory response to concussions is more damaging then the initial impact itself. However, evidence exists for favorable consequences of cytokine production following traumatic brain injuries as well. In some cases, treatments that reduce the inflammatory response will also hinder the brain's intrinsic repair mechanisms. At present, there is no evidence-based pharmacological treatment for concussions in humans. The ability to treat concussions with drug therapy requires an in-depth understanding of the pathophysiological and neuroinflammatory changes that accompany concussive injuries. The use of neurotrophic factors [e.g., nerve growth factor (NGF)] and anti-inflammatory agents as an adjunct for the management of post-concussion symptomology will be explored in this review.

## Introduction

The awareness of the consequences that arise from mild traumatic brain injuries (mTBI), commonly referred to as concussions, has been on the rise and is a “hot topic” in the media, medicine, and basic research. In light of increasing awareness, a clear understanding of the immediate and long-term neural pathology underlying a concussive injury is incomplete thereby precluding both proper assessment and suitable treatment strategies. Because of this, the current review paper will examine the pathophysiological and neuroinflammatory changes that accompany concussive injuries with the hope of improving assessment and treatment to curtail the myriad of neurological and psychological consequences associated with a concussion.

No single definition of concussion (used interchangeably with mTBI) is accepted across all medical or research disciplines, though a number of definitions are available (Comper et al., 2005). In 2001, the first International Symposium on Concussion in Sport was organized (Aubry et al., 2002) and a viable definition to describe concussive injuries was offered by the Concussion in Sport Group. Their definition of a concussion was as follows: “a complex pathophysiological process affecting the brain, induced by traumatic biomechanical forces” (Cantu et al., 2006). The definition was further expanded upon to include the following: “temporary impairment of neurological function that heals by itself over time, and that neuroimaging normally shows no gross structural changes to the brain as the result of the condition” (Anderson et al., 2006). Because this definition was maintained after the 3rd International Conference on Concussion in Sport held in Zurich (McCrory et al., 2009), it will be followed for the purpose of this paper noting that the group provided an update and recommended that the terms concussion and mTBI refer to different injury constructs and should not be used interchangeably (McCrory et al., 2009).

Traumatic biomechanical forces can occur as a result of direct (to the head) or indirect (to the body) impact (e.g., sports, workplace accidents), rapid acceleration or deceleration (e.g., motor vehicle accidents), or intense changes in pressure (e.g., blast exposure). Currently, there is no universally accepted set of symptoms required to be present in order for a patient to be diagnosed with a concussion. Rather, there is a wide range of telltale symptoms that may occur as a consequence of a concussion, which include headache, nausea, vomiting, dizziness, fatigue, abnormal sleeping patterns, drowsiness, and more (Randolph et al., 2009; Ledic et al., 2012; Slobounov et al., 2012). The pursuit for a comprehensive set of defining symptoms remains to be an area of intense investigation.

The prevalence of concussions has dramatically increased over the past decade. This could be due to increased awareness and recognition of symptoms as well as improvements in diagnostic tests and tools, increased survival rates in vehicular accidents and blast victims, as well as the adoption of more liberal concussion criteria. In the general population, there are a reported 545 traumatic brain injury (TBI) cases per 10,000 people. Of these, 88% of cases were determined to be concussions (Andersson et al., 2003; Bazarian et al., 2005). While it is estimated that 25–42% of people who suffer concussions do not report them, concussions still account for a large proportion of documented hospitalizations and impose a 1–2 billion dollar burden on the United States annually (Feinstein and Rapoport, 2000; Goldberg et al., 2007). With such persuasive evidence that concussions are becoming a silent epidemic, it is surprising that there is no effective concussion management system in place.

With an increasing awareness of the deficits produced by concussion, there is an increasing demand for readily available treatments. This has provided the impetus for researchers to explore not only the signs and symptoms but also the neural pathological mechanisms and long-term outcomes associated with concussions. Over the past decade there have been numerous guidelines recommended in an attempt to grade concussions and provide return to play or return to work strategies (Cantu and Register-Mihalik, 2011; Laker, 2011). Along with the rising awareness of the overt signs and symptoms of concussions, there have been momentous advancements in the tools used to properly diagnose concussions (McCullough and Jarvik, 2011). One inherent problem with diagnosing concussions, however, is that presenting symptoms are dependent on personal factors such as age, gender, and history of traumatic brain injuries (Andersson et al., 2003; Field et al., 2003). To further complicate matters, patients, especially athletes, are often reluctant to report any symptoms at all (McCrea et al., 2004; Kutcher et al., 2010). Initial evaluations performed on suspected concussed patients involve a review of the patient's history of head injuries followed by an array of cognitive and behavioral tests in order to make appropriate diagnoses (Putukian, 2011; Scorza et al., 2012). Because there may be changes in neurological function, even in the absence of behavioral abnormalities (Gosselin et al., 2010), there has been a move toward advanced imaging and biomarker-based approaches (Davis et al., 2009; Difiori and Giza, 2010; Jeter et al., 2012). The ability to accurately diagnose concussions has progressed with the use of: postural correlates, MRI, fMRI, EEG, and biomarkers in conjunction with behavioral and neuropsychological tests (Inglese et al., 2005; Bazarian et al., 2007; Pulsipher et al., 2011; Slobounov et al., 2012). These objective neuroimaging and biomarker-based approaches have addressed the issues surrounding the subjectivity of strictly behavioral and self-reported symptoms allowing for more precise detection of neurobiological alterations associated with concussions.

## Neuropathophysiology of concussions

Concussions are the result of biomechanical forces that result in rapid acceleration and decelerations of the brain often leading to functional impairments in the absence of overt structural damage (Fineman et al., 2000; Gurkoff et al., 2006; Prins et al., 2010). The metabolic cascade following this type of injury has been studied extensively in animal models as well as humans (Giza and Hovda, 2001; Barkhoudarian et al., 2011; Giza and Difiori, 2011). Animal models, such as controlled cortical impact (CCI), weight drop, fluid percussion, vacuum deformation, and inertial acceleration have been used considerably to study concussions and advance our understanding of the neural pathology of brain injuries (Giza and Difiori, 2011; O'Connor et al., 2011). Based on an assessment of this work, rotational forces seem to be a prerequisite for producing the diffuse damage in the brain that underlies the signs and symptoms associated with a concussion (Ibrahim et al., 2010). Strong rotational forces cause stretching and shearing of axonal and cell membranes (i.e., diffuse damage), which leads to an unchecked flux of ions through formerly regulated channels and membranes (Farkas et al., 2006). As a consequence of ionic disequilibrium, an indiscriminate release of excitatory neurotransmitters occurs, instigating a widespread, and potentially excitotoxic, neuronal depolarization (Katayama et al., 1990; Kawamata et al., 1992). Furthermore, axonal stretching facilitates the opening of voltage-dependent K^+^ channels causing an abnormal efflux, and ultimately extracellular accumulation of K^+^ (Takahashi et al., 1981; Katayama et al., 1990). In attempt to restore ionic equilibrium, adenosine triphosphate (ATP)-dependent Na^+^/K^+^ pumps work in overdrive, necessitating increased glucose metabolism, which in turn leads to further metabolic dysfunction (Yoshino et al., 1991; Andersen and Marmarou, 1992). This trauma-induced state of hyperglycolysis occurs within minutes and can persist for hours depending on the type and severity of brain injury (Yoshino et al., 1991). Hyperglycolysis leads to the overproduction of lactate, which causes another wave of downstream consequences, ultimately disrupting neuronal functioning (Kalimo et al., 1981; Gardiner et al., 1982). To further complicate matters, over-activation of N-methyl-D-aspartate (NMDA) receptors via hyper-release of glutamate causes a large influx of Ca^2+^ that accumulates in the mitochondria, hindering glucose metabolism leading to impairments in ATP production (Xiong et al., 1997; Babikian et al., 2012). This acute state of hyperglycolysis subsides shortly after the injury and is followed by a prolonged state of hypoglycolysis and reduced cerebral blood flow (Martin et al., 1997). The resulting mismatch in glucose supply vs. demand following a concussion is thought to produce some of the immediate post-concussive symptoms and leave the brain vulnerable to secondary injuries (Giza and Hovda, 2001). For a detailed review on the neurometabolic cascade following a concussion, see Giza and Hovda (2001).

Despite the initial metabolic cascade caused by mechanical forces, much of the damage suffered from a concussion has been attributed to the delayed progression of secondary biochemical events ultimately leading to neuronal dysfunction and, in some cases, cell death. Understanding these delayed secondary events following concussions is crucial for the development of appropriate treatment strategies.

## Inflammatory response to concussions

Initially, it was believed that the brain was incapable of marshaling an inflammatory response to combat infection or disease due to the selective permeability of the blood–brain barrier (BBB). However, it is now well established that neuroinflammation can occur independently of changes in BBB permeability and is commonly seen in response to almost all neurological disorders, including concussions (Csuka et al., 1999, 2000; Fassbender et al., 2000; Morganti-Kossmann et al., 2001; Gentleman et al., 2004; Werner and Engelhard, 2007; Ramlackhansingh et al., 2011; Yu et al., 2011; Kabadi et al., 2012). Interestingly, the permeability of the BBB appears to be altered following events such as combat injury or infection that provoke inflammatory responses (Cortez et al., 1989; Fukuda et al., 1995; Csuka et al., 1999; Morganti-Kossmann et al., 1999). Within the central nervous system (CNS), the inflammatory response involves the recruitment of neutrophils and monocytes to the site of injury in an attempt to aid the injured tissue via the secretion of cytokines and other signaling molecules (Wang and Feuerstein, 2000; Szmydynger-Chodobska et al., 2012). In addition to the recruitment of neutrophils and monocytes, the brain also possesses native cells capable of organizing an inflammatory response themselves. Microglia and astrocytes, for example, express inflammatory mediators and secrete inflammatory cytokines locally (Singh et al., 2011; Galic et al., 2012). Glial fibrillary acidic protein (GFAP) expression is widely used as an estimate of astrocyte activation and has recently been considered as a characteristic marker in brain-injured patients (Schiff et al., 2012). It has been shown that patients who have suffered multiple traumatic injuries, with no history of brain injury, show control levels of GFAP expression, while patients with a history of brain injury show increased GFAP expression as a function of the severity of brain injury (Vos et al., 2010; Yu et al., 2011; Metting et al., 2012; Zurek and Fedora, 2012). Activation of astrocytes and microglia, as evidenced by protracted GFAP expression, results in the production and secretion of inflammatory cytokines (Csuka et al., 2000; Davalos et al., 2005). The injured brain, as is the case following a concussion, undeniably produces an inflammatory response (Holmin et al., 1998). However, the role of the inflammatory response following CNS injury remains unclear. On the one hand, prolonged exposure to inflammatory cytokines is ultimately harmful, shifting the intrinsic neuroprotective efforts of the immune response to the detrimental effects of neuroinflammation (Barone and Parsons, 2000; Bermpohl et al., 2007; Kumar and Loane, 2012). On the other, it has been suggested that neuroinflammation contributes to the neuroprotective regenerating efforts of the brain and in its absence the cumulative damage is increased following injury (Ziebell and Morganti-Kossmann, 2010; Khuman et al., 2011). Here we review the neuroinflammatory response to TBI with a focus on the beneficial and detrimental effects of inflammation following a concussion.

### Interleukin-1 (IL-1)

Following the mechanical injury suffered by concussed patients, there is an acute cytokine response thought to represent, in part, the neuroprotective defense mechanisms of the brain (Scherbel et al., 1999). IL-1, a family of 11 cytokines known for their regulation of inflammatory responses, has been shown to increase rapidly in both human and rodent cases of mild to severe TBI (Woodroofe et al., 1991; Fan et al., 1995; Winter et al., 2002). Within this cytokine superfamily, IL-1α and IL-1β convey a pro-inflammatory response that aids in the defense against infection or injury. In rodent models of concussion, IL-1α and IL-1β are up regulated within hours following injury (Taupin et al., 1993; Shojo et al., 2010; Dalgard et al., 2012). IL-1α shows an acute spike following a concussion, while IL-1β shows a much more gradual increase that is thought to represent a portion of the delayed cytokine response to CNS injury (Holmin et al., 1997). IL-1β levels remain elevated for days following experimental concussions (Dalgard et al., 2012) and show significantly higher levels relative to other pro-inflammatory cytokines (Yu et al., 2011). The elevation in IL-1β levels seen after brain injury appears to be conditional on the severity of the trauma. As such, it has been shown that experimental injuries leading to contusions, mimicking a more potent TBI than a concussion, produce more IL-1β mRNA expression in tissues surrounding the contusion, which lasts up to 6 days following the onset of injury (Holmin et al., 1997).

The rise in IL-1β following a concussion precedes the secretion of ciliary neurotrophic factor (CNTF) and nerve growth factor (NGF), both of which promote the growth and survival of neurons and defend against the instigation of apoptotic pathways (Gadient et al., 1990; Dekosky et al., 1996; Herx et al., 2000). These data suggest that IL-1β provides neuroprotection following brain injury and therefore might be considered part of the regenerative process. Paradoxically, others have shown that chronic inhibition of IL-1β for up to 1 week following a CCI-induced mild/moderate TBI reduces cerebral edema and tissue loss while improving the cognitive outcome by modifying the inflammatory response (Clausen et al., 2009, 2011). These data suggest that prolonged exposure to IL-1β may be associated with neurotoxic effects following a concussion.

Given that IL-1β is capable of stimulating the release of other proinflammatory cytokines, such as tumor necrosis factor-alpha (TNF-α), it is not surprising the IL-1β inhibition results in an atypical inflammatory response following fluid percussion injuries (Woodroofe et al., 1991; Fan et al., 1995). The IL-1β-dependent hypersecretion of other cytokines may produce a toxic inflammatory environment for neurons surrounding the site of injury. Therefore, interrupting IL-1β immediately following the endogenous secretion of neurotrophic factors might prove to be effective in concussion management. However, what remains to be understood is how changes in the severity of brain injury (mild to moderate to severe TBI) alter the expression of IL-β and thus mediate downstream cytokine responses. More studies are required to better characterize the contribution of IL-1β to the inflammatory mechanisms following a concussion and ascertain an injury severity-specific temporal profile of IL-1 secretion.

### Tumor necrosis factor-α (TNF-α)

Like the IL-1 family of cytokines, TNF-α shows a rapid response to experimental brain injury and is considered to be an early mediator of CNS damage (Shojo et al., 2010). Following experimental TBIs, ranging from a mild closed head injury to a more severe lateral fluid percussion injury, TNF-α rises rapidly and peaks within hours, returning to normal levels within 24 h of the injury (Taupin et al., 1993; Shohami et al., 1994; Fan et al., 1996; Knoblach et al., 1999; Bermpohl et al., 2007; Yang et al., 2010; Khuman et al., 2011; Dalgard et al., 2012). Similar to IL-1β, TNF-α appears to play both neuroprotective and neurotoxic roles following brain injury (Ziebell and Morganti-Kossmann, 2010). TNF-α has two known receptors expressed in the CNS: the p55 TNF receptor (TNFR1) and the p75 TNF receptor (TNFR2). Binding of TNF-α to TNFR1 signals for fibroblast growth, endothelial cell activation/adhesion and apoptosis possibly via NF-κB activation (Perry et al., 2001). Binding of TNF-α to TNFR2 appears to be involved in signaling pro-inflammatory responses as well as enhancing TNFR1-mediated apoptosis (Perry et al., 2001). It has been suggested that differential binding of TNF-α to these receptors may explain the differences between its early detrimental and delayed beneficial properties following concussion or more severe cases of TBI (Arnett et al., 2001). Activation of TNFR1 signaling pathways is associated with the pathological effects of TNF-α while activation of TNFR2 is thought to be neuroprotective (Kolesnick and Golde, 1994; Sullivan et al., 1999).

Acutely, TNF-α alters the permeability of the BBB, a well-characterized physiological consequence of concussions (Ramilo et al., 1990; Kim et al., 1992; Tanno et al., 1992; Habgood et al., 2007). Appropriate alterations to BBB permeability may be necessary to regulate the infiltration of blood-born defense mechanisms following a brain injury (Tanno et al., 1992). For example, mice lacking complete functional TNF-α signaling show greater tissue damage, increased BBB permeability, and increased recovery times following both moderate and severe CCI injuries (Scherbel et al., 1999; Sullivan et al., 1999), suggesting that TNF-α is necessary for normal recovery. However, inhibiting TNF-α transcription and bioactivity pharmacologically following a mild closed head injury has been shown to improve neurological outcome and motor function recovery, normalize BBB permeability and decrease edema size, suggesting inhibition of TNF-α activity facilitates recovery (Shohami et al., 1996, 1997). Paradoxically, TNF-α null mice show an early advantage in neurological function after CCI which is lost over time (Scherbel et al., 1999) suggesting that TNF-α signaling may be neurotoxic in the acute stages of TBI. In conjunction with IL-1, TNF-α stimulates the release of NGF from astrocytes, which may explain some of its neuroprotective effects (Gadient et al., 1990; Knoblach and Faden, 1998). It is also possible that TNF-α promotes proliferation of neurons by acting in the subventricular zone (Wu et al., 2000) or through the induction of NFκ-B expression and subsequent activation of anti-apoptotic pathways (Cortez et al., 1989; Shohami et al., 1999). It appears that central blockade of TNF-α following a concussion may prove to be beneficial, while prolonged antagonism could be detrimental.

It is unclear how levels of TNF-α differ as a function of the severity of brain injury. It has been shown that a concussion does in fact cause an increase in TNF-α secretion (Shojo et al., 2010); however, it is uncertain how the increase in TNF-α differs between a concussion and a more severe brain injury. Furthermore, it has been shown that TNF-α acts synergistically with other proinflammatory cytokines (Chao et al., 1995a; Allan and Rothwell, 2001); therefore, a consideration of these types of interactions is also necessary to fully understand the TNF-α contribution to the inflammatory response following a concussion. To address the issue of treating concussions using TNF-α inhibitors, further experiments will be required to supplement our understanding of the time course of toxicity, the signaling pathways important for recovery, and how the severity of injury dictates the extent of TNF-α secretion.

### Interleukin-6 (IL-6)

TNF-α, through modulation of the NF-κB pathway, is a potent stimulator of IL-6 expression (Vanden Berghe et al., 2000). IL-6 acts as both a pro- and anti-inflammatory cytokine (Lenzlinger et al., 2001) and is considered to be a key regulator during the acute phase of the inflammatory response to infections and tissue damage (Kopf et al., 1994). IL-6 has been shown to increase in experimental models of both mild (Shohami et al., 1994; Holmin et al., 1997) and moderate/severe (Woodroofe et al., 1991; Taupin et al., 1993) TBI in rodents and has been detected at high levels for weeks following severe human brain injuries (Kossmann et al., 1995). In mice lacking IL-6, experimental cortical freeze injuries or cytotoxic brain injuries result in increased oxidative stress, decreased cell survival, and lengthened recovery times compared to WT mice, suggesting that in the absence of IL-6, the brain's intrinsic repair mechanisms are deficient (Penkowa et al., 2000, 2003). These data compliment a series of both human brain injury cases and *in vitro* studies, whereby IL-6 secretion leads to elevated production of NGF in astrocytes and suppresses the production of both TNF-α and IL-1β (Kushima et al., 1992; Kossmann et al., 1996; Ley et al., 2011). In addition, IL-6 deficient mice exposed to a closed cortical impact, mimicking a mild TBI, show exaggerated behavioral abnormalities and increased expression of IL-1β 1 h following injury (Ley et al., 2011).

Overexpression of the IL-6 gene, on the other hand, resulted in shortened recovery times, a reduction in genes involved in the control of oxidative stress and reduction in apoptotic pathway gene expression following a mild cortical freeze injury in mice (Quintana et al., 2008). It should be noted, however, that in this particular model, IL-6 overexpression was selectively astrocyte driven (GFAP-IL-6). Furthermore, although this model of GFAP-IL-6 overexpression produced beneficial effects following an acute cortical injury, these mice showed spontaneous neuroinflammatory responses and developed long-term neurological dysfunctions (Campbell et al., 1993; Quintana et al., 2008).

It has not yet been established how IL-6 participates in the secondary neurodegeneration that occurs in concussed patients. The aforementioned cases propose an association between IL-6 and a variety of CNS injuries; however, there has yet to be a study that investigates the relationship between IL-6 and concussion directly. While IL-6 overexpression may have neuroprotective effects in some instances of CNS damage, it would appear that they are location and time specific. If IL-6 exerts its neuroprotective effects through modulation of NGF, TNF-α or IL-1β, then astrocyte driven overexpression, or exogenous administration, must occur at a time when these factors are still malleable.

### Transforming growth factor-β (TGF-β)

The role of anti-inflammatory cytokines in response to concussion is very poorly understood. It would seem intuitive that anti-inflammatory molecules are secreted to counteract the consequences of a propagating inflammatory response. However, anti-inflammatory cytokines serve opposing roles in response to brain injury; some effects are beneficial while others are detrimental. One of the earliest discovered functions of the anti-inflammatory cytokine, TGF-β, was its ability to promote tissue repair by dampening the inflammatory response (Wahl, 1992). TGF-β expression is induced by the presence of inflammatory cytokines and has been shown to form a negative feedback loop by suppressing the production of pro-inflammatory cytokines such as IL-1, IL-6, TNF-α, and IFN-γ (Benveniste et al., 1995; Chao et al., 1995b). Furthermore, TGF-β can disrupt the recruitment of leukocytes to the site of injury through modulation of chemotactic signals (Wahl, 1994). This negative feedback system ensures that the host is protected from proliferating inflammatory attacks. However, the beneficial effects of TGF-β seem to be dependent on its temporal release and concentration. Excessive expression of TGF-β, for example, has not only been shown to hinder the intrinsic repair mechanisms of the brain but also confer a predisposition for the development of serious infections (Wahl, 1992; Lowrance et al., 1994).

Given that TGF-β is secreted in response to inflammatory cytokines, it is of no surprise that expression of TGF-β peaks within 24 h after TBI in human cases (Csuka et al., 1999; Morganti-Kossmann et al., 1999) as well as animal models of mild TBI (Csuka et al., 1999; Morganti-Kossmann et al., 1999; Yu et al., 2011). It has been suggested that TGF-β confers potential short-term beneficial effects following clinical and experimental TBIs by down regulating the inflammatory response; however, in the long-term TGF-β may be detrimental and increase the risk of developing other neurological disorders (Wahl, 1992; Lenzlinger et al., 2001). When considering the therapeutic potential of TGF-β following brain injury, it should be noted that only local administration has been shown to promote tissue repair, while systemic administration results in an immunosuppressive reaction (Mustoe et al., 1987). Additionally, dosages must be titrated carefully given the enhanced vulnerability to infection caused by excessive TGF-β concentrations (Wahl, 1992; Lowrance et al., 1994). More studies need to be conducted to characterize the effects of TGF-β treatment following concussion particularly addressing the issues of timing, concentration, and route of administration in order to isolate its beneficial effects.

### Interleukin-10 (IL-10)

In addition to TGF-β, the other predominant anti-inflammatory molecule involved in the response to brain injury is IL-10. Increased IL-10 secretion from astrocytes and microglia has been shown following a range of CNS injuries from concussions to severe TBI (Csuka et al., 1999; Maskin et al., 2001; Wu et al., 2005; Yu et al., 2011). Presently, the role of IL-10 following concussion is controversial. It has been suggested that IL-10 is neuroprotective as it decreases levels of reactive oxygenated species (Csuka et al., 1999), decreases the expression of several pro-inflammatory cytokines including IL-1 and TNF-α (Knoblach and Faden, 1998; Csuka et al., 1999), and helps suppress further activation of microglia and astrocytes (Kremlev and Palmer, 2005). It should be noted however that the beneficial effects of IL-10 administration are transient and circumstantial. These beneficial effects appear to be dose- and site-specific, requiring pre-treatment in order to confer protection, while the type of injury model also seems to influence the use of IL-10 as a benefactor. In severe human TBI cases, reports have not shown any correlation between IL-10 levels and recovery time (Csuka et al., 1999), while others report that IL-10 expression is associated with negative outcomes (Shiozaki et al., 2005; Kirchhoff et al., 2008). Similarly, animal models of brain injury show mixed results. One model demonstrated marked improvements in recovery from a TBI following IL-10 treatment (Knoblach and Faden, 1998), while others fail to report any beneficial effects of IL-10 treatment following hypoxia-ischemic brain injury (Lyng et al., 2005). With such a wide variety of the type and severity of traumatic brain injuries used in both clinical and experimental research, it is difficult to gauge the role of IL-10 in response to concussion. Consequently, the contributions of IL-10 to the inflammatory response following mild TBI remain to be determined.

## Treatment

As the awareness of and willingness to report concussive symptoms increases in society, the need for effective treatment strategies has gained momentum. Considerable evidence demonstrating the cumulative and potentially fatal effects of experiencing a concussion while still symptomatic from a previous injury (second impact syndrome, SIS) has prioritized treatments that will shorten recovery times and minimize susceptibility (Deford et al., 2002; Tavazzi et al., 2007; Prins et al., 2010). To date, there is no effective treatment for a concussion. Concussion management thus far has focused on prevention (e.g., helmets, rule changes) and the philosophy of “rest, rest, rest” allowing symptoms to subside naturally before clearance to normal activity is approved.

Recently the use of anti-inflammatory drugs for the treatment of concussion has been closely examined. As it is now becoming more evident that lingering neuroinflammatory mechanisms contribute to the secondary damage following a concussion, the use of non-steroidal anti-inflammatory drugs (NSAIDs) seems to be an attractive option. That being said, there is evidence to suggest NSAIDs may not be the best pharmacotherapy for managing the neurobiological factors underlying concussive injuries. It has been shown that chronic treatment with ibuprofen, one of the most commonly used NSAIDs, worsens the cognitive alterations in rodents exposed to an experimental TBI (Browne et al., 2006). Other NSAIDs, such as minocycline, are capable of reducing apoptotic damage in several forms of CNS injury, such as spinal cord injury, but do not show any beneficial effects when examining recovery times from mild TBI (Stirling et al., 2004; Maier et al., 2005). Taken together, these data demonstrate that preventing an inflammatory response to a concussion is not a viable treatment, despite its effectiveness in treating other traumatic injuries occurring in the CNS. Furthermore, this evidence suggests that the concussed brain presents a unique inflammatory signature as opposed to a general inflammatory response that occurs following any CNS injury.

Other drugs designed to target specific pro-inflammatory cytokines have also been used as potential treatment therapies for TBI patients. HU-211, for example, is used as a selective TNF-α inhibitor (Biegon and Joseph, 1995). TNF-α seems to have the highest cytotoxic properties as compared with other cytokines, and therefore HU-211 may be a viable treatment candidate. As such, administration of HU-211 within 24 h of a mild closed head TBI was shown to decrease cell death within the hippocampus and facilitate recovery of motor function in rodents (Shohami et al., 1997). Unfortunately, human trials showed no differences between the drug and placebo group and HU-211 was removed from clinical trials (Maas et al., 2006).

Very recently, there has been particular attention paid to endogenous anti-inflammatory molecules, such as Nesfatin-1. Nesfatin-1 is a peptide secreted from hypothalamic centers in response to inflammation (Bonnet et al., 2009). Administration of Nesfatin-1 has been shown to decrease inflammation and decrease the expression of apoptotic markers within hours of a TBI (Tang et al., 2012). Specifically, Nesfatin-1 has been shown to reduce the expression of pro-inflammatory cytokines such as TNF-α, IL-1β and IL-6, as well as decrease caspase-3 activity following TBI (Tang et al., 2012). The isolation and administration of endogenous anti-inflammatory molecules, such as Nesfatin-1, provides hope for the use of anti-inflammatory drugs in the treatment of concussive injuries. However, it should be noted that the beneficial effects of Nesfatin-1 administration were preceded by 5 consecutive days of peripheral administration and there was no time-response relationship (Tang et al., 2012). Therefore, it remains unclear whether acute, post-concussive treatment with Nesfatin-1 would provide any lasting protection or if a continuous presence of Nesfatin-1 is required as would result from preventative dosing. Further studies are needed to investigate the potential therapeutic effects of peripherally administered endogenous anti-inflammatory agents following concussions.

The failures of NSAIDs and other anti-inflammatory agents in mitigating post-concussive damage highlight the necessity for customized drugs designed for a very specific use. One issue that must be addressed in order to develop relevant treatment strategies for concussed patients is the timing when treatments are most effective. There seems to be a narrow window of opportunity wherein certain drugs can penetrate the BBB (Habgood et al., 2007) and gain access to the site of injury following a concussion. Drug administration must not occur too soon as to interfere with the neuroprotective effects of inflammation, or too late, when the damage is too great to overcome. For example, a drug that permits the acute secretion of IL-1β and IL-6 but antagonizes any long-term accumulation of these cytokines could potentially harness the early advantages described in the literature while blocking the long-term detrimental effects. In addition, dampening the acute secretion of TNF-α and TGF-β may prove to be beneficial whereas prolonged interruption may lead to deleterious outcomes. Therefore, drug therapies that can strike the balance between acute and delayed actions of cytokines may prove to be appropriate targets for pharmacological treatment of concussion. One issue that remains unclear, however, is whether or not the dueling role of inflammatory cytokines are the consequence of concentration gradients or synergistic effects in the presence of other cytokines secreted at different time points.

## Conclusion

The awareness and recognition of concussive brain injuries is on the rise in large part due to improvements in diagnostic/assessment techniques, increased survival rates from accidents and media attention. With a large number of available definitions, there is no doubt that the term “concussion” will be used in common parlance to describe an array of cognitive symptoms that result from biomechanical forces to the brain. Nevertheless, the search for a viable concussion treatment is higher now than ever before. A further understanding of the detrimental neurobiological effects caused by a concussion will be required in order to develop appropriate treatment strategies. The acute metabolic cascade following a concussion has been characterized in detail over the past several decades (Giza and Hovda, 2001; Barkhoudarian et al., 2011). Unfortunately, targeting the metabolic cascade has failed to produce viable treatment options given its rapid onset and the temporal reality of reaching a concussed patient in time to stop this cascade from progressing. Therefore, treatment strategies focused on managing the neuroinflammatory responses to concussion may prove to be more tangible targets, as it is hypothesized these delayed secondary effects may be the primary source of long-term neural damage. There are promising data to suggest that manipulating neuroinflammation can be used as treatment strategies to manage the long-term deficits produced by a concussive injury. However, the use of general anti-inflammatory drugs will not serve as a “magic bullet” for this silent epidemic. Rather, it seems that a tailored array of pro- and anti-inflammatory compounds given at particular temporal intervals will likely be implemented given the complexity of the inflammatory response to concussion. Treatments will likely differ based on severity of brain injury, age of the patient, and previous history of brain injury. Furthermore, treatment strategies require close attention be paid to when a patient consults a medical professional after brain injury given the fluctuations of inflammatory profiles over time following a concussion.

### Conflict of interest statement

The authors declare that the research was conducted in the absence of any commercial or financial relationships that could be construed as a potential conflict of interest.
